# Single-Nucleotide Polymorphism Masking

**Published:** 2008

**Authors:** Nicole A.R. Walter, Shannon K. McWeeney, Sandra T. Peters, John K. Belknap, Robert Hitzemann, Kari J. Buck

**Keywords:** expression, genome, genetic analysis, microarray analysis, singular nucleotide polymorphism (SNP), SNP masking, mouse genome, laboratory mice, animal models

As described in other articles in this Special Section, microarrays are widely used to evaluate gene expression at the genome scale. However, all too often the importance of data analysis at the level of the individual probe is overlooked. This is a particular problem when trying to detect differences in gene expression levels among genetically unique animals, across inbred animal strains, or among genetically modified animals. Of particular concern is the presence of small modifications in the DNA (i.e., single nucleotide polymorphisms [SNPs]) that occur naturally and differentiate one individual from the next. This article describes the potential impact of SNPs on analyses of gene expression differences and introduces an approach called SNP masking, which implements removal of SNP-affected probes. SNP masking is a valuable and feasible approach that can ameliorate these hybridization problems.

## Impact of SNPs on Gene Expression Analyses

Recent research projects to determine the exact sequence of the mouse genome and other research efforts have identified millions of SNPs in the mouse genome. In all, more than 10 million SNPs now are known to exist among mouse strains. Even for the two strains most commonly used for genetic analyses (i.e., C57BL/6J [B6] and DBA/2J [D2]), approximately 4 million known SNPs exist. Walter and colleagues (2007) recently illustrated the impact that these SNPs have on hybridization studies such as microarray analyses, in which thousands of short pieces of mouse DNA simultaneously are used as probes to determine the expression levels (i.e., the levels of messenger RNA [mRNA]^1^) of certain genes in a sample prepared by the researcher, or in this case, to look for differences in gene expression between two mouse strains. These probes can be very short, with a microarray containing many probes from one gene. Each set of probes is combined in analysis as a probe set, and a microarray will often include several probe sets per gene or transcript. In the experiments, the mRNAs studied bind to (i.e., hybridize with) corresponding probes on the microarray, resulting in a signal that can be read by a special detector and which serves as an indicator of the corresponding gene expression level in the sample.

Walter and colleagues (2007), using computer calculations based on the known locations of probes versus the known SNPs locations, determined which of the probes on a commonly used microarray spanned known SNPs between B6 and D2 mice. Probes including SNPs or sequence mismatches could influence hybridization and therefore cause incorrect detection of the expression level of the genes. This approach identified 13,292 probes on the array that included at least one known SNP and which affected a total of 6,590 probe sets (i.e., approximately 16 percent of the entire array). The presence of these SNP sequences can have a great impact on the interpretation of the experimental results. Thus, if a sample is derived from the same mouse strain as the probes on the microarray and therefore matches the sequence found on the microarray, the experiment will yield a true result (i.e., indicate the true level of expression of the corresponding gene based on hybridization to the probe). If, however, the sample is derived from another strain and contains an alternative form of the probe sequence, the experiment could yield a false result (i.e., indicate a lower level of gene expression simply because of the mismatch in sequence affecting the hybridization of the mRNA to the probe).

**Figure 17:**
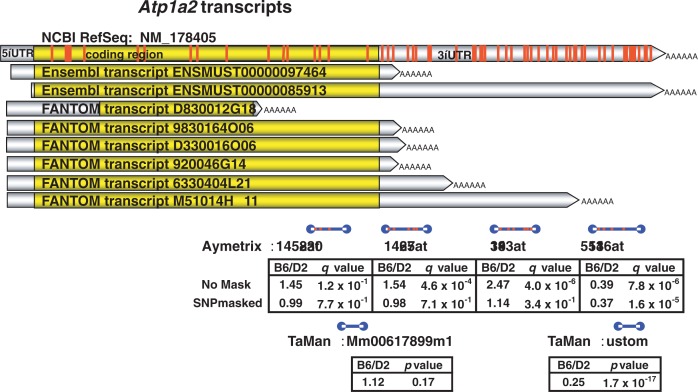
The *Atp1a2* gene generates multiple transcripts (yellow bars) that span a total of 61 known single-nucleotide polymorphisms (SNPs) (shown as red bars) between the C57BL/6J (B6) and DBA2/J (D2) strains. Blue bars represent four Affymetrix microarray probe sets (all of which span known SNPs) and two TaqMan real-time PCR probes (neither of which spans known SNPs). The SNPs confound interpretation of microarray expression results as the Affymetrix gene expression ratio (B6/D2) values differ dramatically depending on whether an SNP mask is applied (i.e., whether the SNP-affected probes are removed from the analyses or not). The masked Affymetrix microarray results are confirmed by TaqMan quantitative real-time PCR results.

Using two different analytic methods, the researchers estimated that these experiments could yield false-positive results in 36 percent and 22 percent of cases, respectively, and false-negative results in 13 percent and 12 percent of cases, respectively (Walter et al. 2007). These findings demonstrate a dramatic lack of concordance in differential expression results owing solely to the presence of SNPs in microarray probes. The number of SNPs that are found in a particular transcript can vary greatly; thus, in some cases only one SNP located in the corresponding probe set will yield a false-positive or false-negative result; in other cases, however, such as that of a gene called *Atp1a2*, 61 SNPs were identified that affected many probes within multiple probe sets for the gene, causing multiple false-positive results (see figure 17).

## Conclusions

Researchers over the years have identified millions of SNPs in the mouse genome alone, and there are many more as-yet-unidentified SNPs that also could affect gene expression probes in hybridization-based assays. Even for the well-studied B6 versus D2 strains, it is estimated that there are many more SNPs than have been described so far. Therefore, it is of utmost importance that investigators performing microarray analyses identify false-positives and false-negatives before carrying out costly followup confirmation experiments on their samples. With the promise of being able to sequence an entire genome for only $1,000 on the horizon, determination of the complete genome sequence of many mouse strains in the near future will provide additional momentum to the field, allowing for complete SNP masking of genetic differences among strains.

## Footnotes

1 mRNA molecules often also are referred to as “transcripts” because they are generated from the DNA template in a process known as transcription.
